# Predictors of cognitive changes in patients with schizophrenia undergoing electroconvulsive therapy

**DOI:** 10.1371/journal.pone.0284579

**Published:** 2023-05-09

**Authors:** Arvind Rajagopalan, Kenny Wai Kwong Lim, Xiao Wei Tan, Donel Martin, Jimmy Lee, Phern-Chern Tor

**Affiliations:** 1 Department of Mood and Anxiety, Institute of Mental Health, Singapore, Singapore; 2 School of Psychiatry, Black Dog Institute, University of New South Wales, Sydney, Australia; 3 Department of Psychosis, Institute of Mental Health, Singapore, Singapore; 4 Lee Kong Chian School of Medicine, Nanyang Technological University, Singapore, Singapore; Istanbul Bakirkoy Prof Dr Mazhar Osman Ruh Sagligi ve Sinir Hastaliklari Egitim ve Arastirma Hastanesi, TURKEY

## Abstract

**Introduction:**

Previous studies on the effects of electroconvulsive therapy (ECT) on cognition in schizophrenia have been inconclusive. This study aimed to identify factors that may predict cognitive improvement or deterioration in patients with schizophrenia after-ECT.

**Materials & methods:**

Patients with schizophrenia or schizoaffective disorder with predominantly positive psychotic symptoms, who were treated with ECT at the Institute of Mental Health (IMH), Singapore, between January 2016 and January 2018, were assessed. Montreal Cognitive Assessment (MoCA), Brief Psychiatric Rating Scale (BPRS) and Global Assessment of Function (GAF) were performed before and after ECT. Patients with clinically significant improvement, deterioration or no change in MoCA scores were compared on demographics, concurrent clinical treatment and ECT parameters.

**Results:**

Of the 125 patients analysed, 57 (45.6%), 36 (28.8%) and 32 (25.6%) showed improvements, deterioration and no change in cognition respectively. Age and voluntary admission predicted MoCA deterioration. Lower pre-ECT MoCA and female sex predicted MoCA improvement. Patients showed improvements in GAF, BPRS and BPRS subscale scores on average, except for the MoCA deterioration group, who did not show statistically significant improvement in negative symptom scores. Sensitivity analysis showed that nearly half the patients (48.3%) who were initially unable to complete MoCA pre-ECT were able to complete MoCA post-ECT.

**Conclusions:**

The majority of patients with schizophrenia demonstrate improved cognition with ECT. Patients with poor cognition pre-ECT are more likely to see improvement post-ECT. Advanced age may be a risk factor for cognitive deterioration. Finally, improvements in cognition may be associated with improvements in negative symptoms.

## 1. Introduction

The impact of Electroconvulsive Therapy (ECT) on cognition has been the subject of much debate [1]. Prior studies identified that variation in the type of ECT and electrode placements could modify the effects of ECT on cognition, Older sine-wave ECT had a greater adverse impact on memory compared to newer techniques using square wave stimulus, ultrabrief pulse width and unilateral electrode placement [2, 3]. Earlier studies on the effects of ECT on cognition was done on subjects with depression. As a core diagnostic symptom of depression is impairment in the ability to think and concentrate, the effect of ECT on cognition may be confounded by disease factors in these earlier studies, as cognition would improve with the abatement of depressive symptoms [4, 5]. Another study found that the cognitive adverse effects were short-lived. Patients followed up at one month and six months after ECT were found to perform as well or even better at cognitive tasks compared to their performance before treatment [6]. Later studies have echoed the above findings and have gone on to compare different parameters of ECT and their impact on cognition specifically, concluding that while there is a risk for cognitive impairment with any form of ECT, sine-wave ECT and bilateral electrode placement are more strongly associated with this than brief-pulse ECT and unilateral electrode placement respectively [7, 8]. Treatment-related factors such as higher dose and increased frequency of ECT and patient-related factors such as advanced age and pre-existing brain diseases are also associated with deterioration of cognition [1, 9].

Cognitive deficits are a core feature of schizophrenia with impact on social occupational functioning. A review has found that cognitive deficits in schizophrenia appear distinct from positive and negative symptoms, with disorganization factors having the strongest association with cognitive test scores. Deficits in social cognition are also present in individuals with schizophrenia and impact functioning [10].

The effects of ECT on cognition for the treatment of schizophrenia are unclear. Older studies have been inconclusive. Some found subtle memory impairments in patients after ECT [11] whilst others found no memory impairment at all [12]. The Cochrane Review of ECT in schizophrenia concluded that the use of ECT seems to be associated with greater cognitive impairment immediately post-ECT when compared to conventional antipsychotics and this impairment appears to be transient. The review acknowledged that this conclusion is informed by only a small number of studies with small sample sizes [13]. More recent evidence suggests that ECT either has no significant effect on cognition, or may even improve cognition, in patients with schizophrenia. A small two-year follow-up study of nine adolescents with schizophrenia undergoing ECT, matched to nine controls, found no significant differences in cognitive scores (assessed using the Neuropsychological Assessment Scale) between the two groups at the two-year end-point [14]. Similar results were found in a study comparing 10 patients on maintenance ECT for schizophrenia to matched controls. However, the study was limited by its small sample and cognition was measured at least 4 months after the course of ECT [15]. More recently, a study of 31 adults inpatients with treatment-resistant schizophrenia undergoing adjunctive ECT found that ECT may have positive effects on some aspects of cognition, such as immediate and delayed verbal memory and executive functioning. ECT had probable positive effects on visual memory and psychomotor speed, however this was no longer statistically significant after correction for multiple testing [16]. Our group previously compared Montreal Cognitive Assessment (MoCA) scores for patients with schizophrenia before and after ECT for four different modalities of ECT. We found that MoCA delayed recall scores improved in general. When stratified across the different modalities, delayed recall improved with seizure threshold-based dosing but worsened with age-based dosing [17].

The possibility that ECT may improve cognition in patients with schizophrenia is promising which merits further study. Understanding clinical factors that could predict improvement or deteriorations in cognition would guide clinicians in the selection of patients who would benefit most from ECT in schizophrenia. As such, we conducted an exploratory study to investigate if there are patient-related or ECT-related factors could predict improvements or deteriorations in cognition in this population.

## 2. Materials and methods

### 2.1 Study setting & participants

This is a retrospective cohort study among patients who had been referred to the ECT service at the Institute of Mental Health, Singapore between January 2016 and January 2018. Patients were referred to the ECT service either due to clinical assessment by treating psychiatrist to have poor response to drug treatment or other reasons such as catatonia, patient preference, intolerable side effects to medications or severe symptoms. Most patients would have failed at least 1–2 months of inpatient treatment. We included patients with a clinical diagnosis of schizophrenia or schizoaffective disorder, with predominant positive psychotic symptoms based on DSM-IV/V or ICD-10. Patients with schizoaffective disorder with depressive or manic symptoms based on clinician evaluation were excluded from analysis. We also excluded patients who did not complete either pre- or post-ECT MoCA assessment. Ethics approval for this study was provided by the National Healthcare Group’s Domain Specific Review Board and a waiver of informed consent was obtained as de-identified data was taken from a clinical registry database.

### 2.2 Electroconvulsive therapy

ECT was delivered using a Thymatron system IV device (Somatics, USA) or MECTA SpECTrum 5000Q device (MECTA, USA) with handheld electrodes. Each patient’s empirically determined seizure threshold was used for individualized dosing. ECT was administered via bitemporal (BT), bifrontal (BF) or right unilateral (RUL) electrode positioning. BT ECT was delivered at 0.5ms pulse width at 1.5x dose relative to seizure threshold (DRST), BF ECT was delivered at 1.0ms pulse width at 1.5xDRST and RUL ECT was delivered at 0.5ms pulse width at 5xDRST. Propofol (1mg/kg) and succinylcholine (0.5mg/kg) were used for anaesthesia and muscle relaxation respectively.

### 2.3 Data collection and ECT outcome assessment

Patients’ medical registration data was collected using the Clinical Alliance and Research in ECT (CARE) framework [18]. Baseline information of patient’s socio-demographics and concurrent pharmacological treatments, i.e., antidepressant, antipsychotic, mood stabilizer and stimulant usage were noted.

Patients were assessed with the Brief Psychiatric Rating Scale (BPRS) [19], Global Assessment of Function (GAF) [20] and MoCA [21, 22] prior to the initiation of ECT and after the 6^th^ and 12^th^ ECT sessions (typically 2 days after the session). Alternate versions of the MoCA were used in the post-ECT tests to minimise practice effect. The latest available score was used as the post-ECT scores in the analyses. BPRS ratings were completed by two medical officers trained in the delivery of ECT and who also underwent BPRS training using standardized training videos under the supervision of a senior psychiatrist. Intra-class correlation between the two medical officers and the senior psychiatrist was 0.77 and 0.87, respectively. Higher BPRS scores indicate poorer psychiatric condition. Individual BPRS subscale scores were also calculated [23]. The MoCA was administered by registered nurses, who were trained and certified in the administration of the MoCA. Singapore is a multi-racial country with a resident population comprising of 74.3% ethnic Chinese, 13,5% Malay and 9% Indians [24]. In our study MoCA was administered in the patients’ preferred language, either English or in validated translation for Mandarin, Malay or Tamil languages [25–27]. Higher MoCA total scores indicate better cognition function. The GAF scale was used to assess overall functioning in patients with higher scores indicating better functioning.

### 2.4 Statistical analysis

Sociodemographic and clinical characteristics were described. Normally distributed continuous variables were presented as mean ± standard deviation (SD), skewed distributed continuous variables were presented as median/interquartile range (IQR) and categorical variables as count and percentage (%).

BPRS, MoCA and GAF scores at pre-ECT and post-ECT treatment were compared using paired t tests. Multinomial regression was conducted to identify the potential risk factors associated with MoCA groups. This was done by splitting the sample population into those with an increase of at least 2 points in post-ECT MOCA compared to pre-ECT MOCA as “MoCA improvement”, those with a reduction of at least 2 points in post-ECT MOCA compared to pre-ECT MOCA as “MoCA deterioration” and those with 1 point or no change in MoCA post-ECT compared to pre-ECT as “MoCA no change” [28]. Covariates included in the regression model were patients’ age, sex, number of previous episodes, medication class prescribed, number of failed medication trials, past ECT treatment, mean propofol dosage, mean ECT dosage and mean electroencephalogram (EEG) score [29]. These covariates were selected based on prior studies [30–32]. For sensitivity analysis, we removed the outliers (> or < mean±2.5 SD) of MoCA readings.

All statistical analyses were conducted using IBM SPSS Statistics, Version 22.0 (Armonk, NY: IBM Corp). Statistical significance was set at *p*<0.05.

## 3. Results

A total of 125 patients diagnosed with schizophrenia or schizoaffective disorder with predominant positive symptoms were included in the analyses. The baseline demographic and clinical characteristics of the study population are summarised in Table 1.

**Table 1:** Baseline demographics and clinical characteristics.

Patient characteristics			Total (n = 125)		MOCA group					
					MOCA no change (n = 32, 25.6%)		MOCA improvement (n = 57, 45.6%)		MOCA deterioration (n = 36, 28.8%)	
Age	≤ 55 years		102	81.6%	29	90.6%	47	82.5%	26	72.2%
	> 55 years		23	18.4%	3	9.4%	10	17.5%	10	27.8%
Number of ECT sessions (mean±SD)			9.9±2.9		10.3±2.6		10.0±3.0		9.4±3.1	
Sex (n, %)		Female	71	56.8%	11	15.5%	39	54.9%	21	29.6%
		Male	54	43.2%	21	38.9%	18	33.3%	15	27.8%
Admission status (n, %)		Involuntary	88	70.4%	24	27.3%	45	51.1%	19	21.6%
		Voluntary	37	29.6%	8	21.6%	12	32.4%	17	45.9%
Consent * (n, %)		Consent by others	105	84%	24	22.9%	49	46.7%	32	30.5%
		Own consent	17	13.6%	7	41.2%	6	35.3%	4	23.5%
Number of previous episodes (n, %)		>3	86	68.8%	17	19.8%	41	47.7%	28	32.6%
		1–3	35	28%	12	34.3%	15	42.9%	8	22.9%
		0	4	3.2%	3	75.0%	1	25.0%	0	0.0%
Antidepressants (n, %)		YES	35	28%	12	34.3%	12	34.3%	11	31.4%
		NO	90	72%	20	22.2%	45	50.0%	25	27.8%
Antipsychotics other than clozapine (n, %)		YES	116	92.8%	28	24.1%	52	44.8%	36	31.0%
		NO	8	6.4%	4	50.0%	4	50.0%	0	0.0%
Lithium (n, %)		YES	10	8%	3	30.0%	6	60.0%	1	10.0%
		NO	115	92%	29	25.2%	51	44.3%	35	30.4%
Benzodiazepines (n, %)		YES	69	55.2%	16	23.2%	33	47.8%	20	29.0%
		NO	56	44.8%	16	28.6%	24	42.9%	16	28.6%
Anticonvulsants (n, %)		YES	25	20%	9	36.0%	12	48.0%	4	16.0%
		NO	100	80%	23	23.0%	45	45.0%	32	32.0%
Failed Antipsychotics (n, %)		≥3	79	63.2%	21	26.6%	33	41.8%	25	31.6%
		1–2	44	35.2%	10	22.7%	23	52.3%	11	25.0%
		None	2	1.6%	1	50.0%	1	50.0%	0	0.0%
Clozapine (n, %)		YES—with no/minimal response	33	26.4%	5	21.7%	11	47.8%	7	30.4%
		YES—with partial/good response	15	12%	3	20.0%	8	53.3%	4	26.7%
		NO	86	68.8%	24	27.9%	38	44.2%	24	27.9%
ECT type (n, %)		BT	21	16.8%	8	38.1%	10	47.6%	3	14.3%
		RUL	6	4.8%	0	0.0%	4	67%	2	32%
		BF	97	77.6%	24	24.7%	43	44.3%	30	30.9%

Key:

*—Missing data, ECT–Electroconvulsive therapy, BT–Bitemporal, RUL–Right Unilateral, BF—Bifrontal

The average age was 39.9±15.2 (mean±SD) years and 56.8% were female. The majority of ECT treatment type was Bifrontal (BF) ECT [N = 97 (77.6%)]. The mean number of ECT sessions was 10. 88 patients without post-ECT MoCA scores were excluded from the analysis. In addition, 60 patients without pre-ECT MoCA scores were excluded as well, as they were too ill to be assess cognitively. However, 29 (48.3%) of them were able to complete MoCA post-ECT, suggesting an ECT associated improvement in cognition, as they were not able to participate in a MoCA assessment initially. Table 2 summarises the outcome assessments pre- and post-ECT stratified by cognition group. Analysed by paired t test, there was a global improvement of BPRS, MoCA and GAF scores after ECT treatment. Mean BPRS score decreased from 49.44 to 34.30 after ECT treatment (P<0.001), mean MoCA improved from 19.62 to 22.17 after ECT treatment (P 0.003) and mean GAF score increased from 42.43 to 58.55 after ECT treatment (P<0.001)—BPRS subscale scores improved across MoCA groups except for the negative symptom subscale score, which displayed no significant change in the MoCA deterioration group.

**Table 2:** Outcome assessments before and after ECT treatment stratified by cognition group.

ECT outcome assessment	Total				MoCA group											
					MOCA no change (n = 32)				MOCA improvement (n = 57)				MOCA deterioration (n = 36)			
	Mean	SD	N	P-value (pre vs post-ECT)	Mean	SD	N	P-value (pre vs post-ECT)	Mean	SD	N	P-value (pre vs post-ECT)	Mean	SD	N	P-value (pre vs post-ECT)
Pre-ECT BPRS total score	49.44	11.07	120	<0.001**	47.13	8.74	32	<0.001**	50.81	11.53	53	<0.001**	49.49	12.17	35	<0.001**
Post-ECT BPRS total score	34.30	7.34	92		33.00	5.96	23		34.40	7.32	42		35.26	8.45	27	
Pre-ECT psychotic symptoms	13.51	5.080	108	<0.001**	11.71	4.23	31	<0.001**	14.78	4.98	45	<0.001**	13.47	5.57	32	<0.001**
Post-ECT psychotic symptoms	7.96	4.168	97		7.68	3.51	25		8.31	4.41	45		7.63	4.40	27	
Pre-ECT negative symptoms	7.83	3.632	115	<0.001**	8.14	2.84	29	<0.001**	8.00	4.18	52	0.014*	7.32	3.36	34	1.000
Post-ECT negative symptoms	6.56	3.448	107		5.41	2.61	27		6.58	2.81	50		7.57	4.67	30	
Pre-ECT depressive symptoms	7.97	3.946	101	<0.001**	8.29	4.25	31	0.005*	7.90	3.79	42	0.001*	7.71	3.95	28	0.020*
Post-ECT depressive symptoms	5.84	2.433	93		5.84	1.82	25		5.82	2.62	44		5.88	2.72	24	
Pre-ECT manic symptoms (activity subscale) (4–28)	6.82	3.077	115	<0.001**	5.84	1.82	32	0.003*	7.28	3.55	50	0.001*	7.06	3.15	33	0.009*
Post-ECT manic symptoms	4.92	1.779	100		4.64	1.11	25		5.09	2.03	47		4.89	1.83	28	
Pre-ECT MOCA	19.62	8.24	125	0.003*	24.09	7.58	32	0.500	15.35	7.27	57	<0.001**	22.39	7.02	36	<0.001**
Post-ECT MOCA	22.17	6.89	125		24.00	7.51	32		24.12	4.69	57		17.44	7.13	36	
Pre-ECT GAF	42.43	7.04	115	<0.001**	44.48	4.08	29	<0.001**	41.67	7.33	51	<0.001**	41.83	8.28	35	<0.001**
Post-ECT GAF	58.55	7.99	87		58.81	5.68	21		58.84	9.26	38		57.96	7.83	28	

Key: ECT–Electroconvulsive Therapy, GAF–Generalised Assessment of Function, BPRS–Brief Psychiatric Rating Scale, MoCA–Montreal Cognitive Assessment

*P <0.05

** P <0.001

In the multinominal regression model, age >55 was significantly associated with MoCA deterioration after adjustment for sociodemographic and clinical characteristics [adjusted β:2.13, 95% CI:1.13–62.54, p = 0.037]. Involuntary admission was not associated with less MoCA deterioration before adjustment but showed associations after adjustment [adjusted β: -1.87, 95% CI:0.03–0.79, p = 0.025]. A lower pre-ECT MoCA was associated with MoCA improvement both before [crude β: -0.16, 95% CI:0.79–0.92, p<0.001] and after [adjusted β: -0.15, 95% CI:0.78–0.94, p<0.002] adjustment. Female sex was associated with MoCA improvement both before [crude β:1.42, 95% CI:1.65–10.37, p = 0.002] and after adjustment [adjusted β:2.75, 95% CI:2.61–94.10, p = 0.003]. pre-ECT GAF, BPRS scores, number of ECTs, consent and co-administered psychotropics were not predictive of MoCA deterioration or improvement. See Tables 3 and 4.

**Table 3:** Predictors of MOCA deterioration in ECT.

Outcome	Risk predictor		Crude						Adjusted				
			B	OR	95% Cl for OR		P value	B		OR	95% CI for OR		P value
					Lower Bound	Upper Bound					Lower Bound	Upper Bound	
MOCA deterioration vs MOCA no change	Age	> 55 years	0.72	2.06	0.52	8.10	0.302	2.13		8.42	1.13	62.54	**0.037** *
		≤ 55 years	ref.										
	No. ECT		-0.11	0.89	0.75	1.06	0.183	-0.22		0.80	0.58	1.10	0.172
	MoCA pre-ECT		-0.05	0.96	0.88	1.03	0.265	0.02		1.02	0.91	1.13	0.769
	GAF pre-ECT		-0.07	0.93	0.86	1.02	0.111	-0.04		0.96	0.84	1.11	0.61
	BPRS pre-ECT		0.02	1.02	0.98	1.07	0.375	0.00		1.00	0.92	1.08	0.923
	Sex	Female	0.98	2.67	1.00	7.16	0.051	1.37		3.93	0.95	16.34	0.060
		Male	Ref.										
	Admission status	Involuntary	-0.99	0.37	0.13	1.05	0.061	-1.87		0.15	0.03	0.79	**0.025** *
		Voluntary	Ref.										
	Consent	By others	0.85	2.33	0.61	8.89	0.214	1.24		3.46	0.36	33.48	0.285
		By self	Ref.										
	Antidepressants	YES	-0.31	0.73	0.27	2.01	0.546	-0.34		0.71	0.11	4.39	0.711
		NO	Ref.										
	Lithium	YES	-1.29	0.28	0.03	2.80	0.276	-2.83		0.06	0.00	1.61	0.093
		NO	Ref.										
	Benzodiazepines	YES	0.22	1.25	0.48	3.25	0.647	1.35		3.86	0.91	16.48	0.068
		NO	Ref.										
	Anticonvulsants	YES	-1.14	0.32	0.09	1.17	0.084	-0.36		0.70	0.09	5.76	0.740
		NO	Ref.										
	Clozapine	YES—with no/minimal response	0.34	1.40	0.39	5.03	0.606	1.12		3.05	0.40	23.14	0.280
		YES—with partial response	0.29	1.33	0.27	6.61	0.725	-0.54		0.58	0.05	6.20	0.655
		NO	Ref.										

Abbreviations: ECT–Electroconvulsive Therapy, GAF–Generalized Assessment of Function, BPRS–Brief Psychiatric Rating Scale, MoCA–Montreal Cognitive Assessment

*P<0.05

**Table 4:** Predictors of MoCA improvement in ECT.

Outcome	Risk predictor		Crude					Adjusted				
			B	OR	95% Cl for OR		P value	B	OR	95% CI for OR		P value
					Lower Bound	Upper Bound				Lower Bound	Upper Bound	
MOCA improvement vs MOCA no change	Age	> 55 years	1.31	3.72	0.92	15.00	0.065	0.94	2.56	0.34	19.28	0.362
		≤ 55 years	Ref.									
	No. ECT		-0.04	0.96	0.82	1.12	0.619	-0.19	0.83	0.62	1.10	0.191
	MoCA pre-ECT		-0.16	0.85	0.79	0.92	**<0.001** **	-0.15	0.86	0.78	0.94	**0.002** *
	GAF pre-ECT		-0.07	0.93	0.86	1.01	0.081	-0.12	0.89	0.78	1.01	0.079
	BPRS pre-ECT		0.03	1.03	0.99	1.08	0.139	0.05	1.05	0.97	1.13	0.243
	Sex	Female	1.42	4.14	1.65	10.37	**0.002** *	2.75	15.67	2.61	94.10	**0.003** *
		Male										
	Admission status	Involuntary	0.22	1.25	0.45	3.48	0.669	-0.26	0.77	0.12	4.90	0.784
		Voluntary	Ref.									
	Consent	By others	0.87	2.38	0.72	7.87	0.155	-0.82	0.44	0.04	5.14	0.512
		By self										
			Ref.									
	Antidepressants	YES	-0.81	0.44	0.17	1.16	0.097	0.14	1.15	0.20	6.74	0.875
		NO	Ref.									
	Lithium	YES	0.13	1.14	0.26	4.89	0.863	-0.64	0.53	0.05	5.09	0.581
		NO	Ref.									
	Benzodiazepines	YES	0.32	1.37	0.58	3.28	0.473	0.32	1.37	0.30	6.18	0.682
		NO	Ref.									
	Anticonvulsants	YES	-0.38	0.68	0.25	1.85	0.452	0.85	2.33	0.37	14.75	0.368
		NO	Ref.									
	Clozapine	YES—with no/minimal response	0.33	1.39	0.43	4.50	0.583	1.52	4.59	0.62	33.72	0.134
		YES—with partial response	0.52	1.68	0.41	6.98	0.472	0.58	1.79	0.23	14.03	0.578
		NO	Ref.									

Abbreviations: ECT–Electroconvulsive Therapy, GAF–Generalized Assessment of Function, BPRS–Brief Psychiatric Rating Scale, MoCA–Montreal Cognitive Assessment

*P<0.05

** P<0.001

To account for possible insignificant readings from the patients’ MoCA scores, the analyses were done again excluding outliers of MoCA values, which were more than 2.5 standard deviations from the mean. The multinomial regression model after removing the outliers further confirmed our findings. The results are shown in S1, S2 Tables.

## 4. Discussion

To our knowledge, this study is the first to compare the characteristics of patients with either improved or deteriorated cognition receiving ECT for schizophrenia or schizoaffective disorder with predominant positive psychotic symptoms. We found that majority of patients showed improved cognition post-ECT. Additionally, patients with lower baseline cognition, as indicated by lower MoCA scores, seem to show a greater improvement in MoCA with ECT, supporting findings from a previous study on geriatric patients undergoing ECT [33].

Literature suggests that the cognitive impairment seen in schizophrenia is independent of the burden of psychotic symptoms, with the evidence showing no associations between positive and cognitive deficits. Heydebrand et al found that there is a relationship between negative symptoms and cognitive function in first episode psychosis but it only accounts for a minor portion after controlling for potential confounders [34]. This has led to the suggestion that cognitive impairment could be an independent treatment target in schizophrenia [35]. To date, only one previous study has investigated factors associated with cognitive improvement for patients with schizophrenia undergoing ECT [36]. This study found that pre-ECT MoCA was a significant predictor of post-ECT MoCA change; patients with clinically significant (≥2 point) improvement in MoCA had significantly lower pre-ECT MoCA scores than those who did not show improvement and conversely, those who with a clinically significant deterioration in MoCA had significantly higher pre-ECT MoCA scores. MoCA improvement was independent of changes in psychotic symptoms. Our study similarly found that lower pre-ECT MoCA strongly predicted MoCA improvement with ECT, also independent of changes in BPRS. Unlike the previous study, our study did not find that a higher pre-ECT MoCA predicted deterioration in MoCA scores. It is possible that this represents a return to normal cognitive functioning in patients with underlying impaired cognition undergoing ECT, as opposed to a direct effect of ECT on cognition. Nevertheless, it suggests that poor pre-treatment cognition should not be a contraindication to prescribing ECT for these patients.

Patients with deterioration in MoCA did not show an improvement in negative symptom scores, unlike those with improvement or no change in MoCA. A previous study on patients with psychotic depression found that improvements in cognition were associated with improvements in negative symptoms [37]. It is possible then that improvements in cognition in schizophrenia are similarly related to improvements in negative symptoms with ECT as described in Heydebrand et al. [34]. This relationship is intriguing and merits further study to further elucidate the pathways negative symptoms contribute to cognition.

Early studies of the correlation between age and cognitive change in patients with depression undergoing ECT found that symptom burden as well as cognition, measured using the Mini Mental State Examination (MMSE), improved with increasing age [38]. These findings were corroborated by a more recent study, which found statistically significant improvement in MMSE scores for elderly patients with depression after ECT, compared to younger patients who did not show statistically significant improvement [39]. A 2006 study of elderly patients undergoing ECT for depression studied changes in multiple cognitive domains, and found that ECT improved cognition in these patients independently of the reduction in depressive symptoms [40]. However, there are studies that contradict these findings. A large prospective multicentre trial found that advanced age was associated with poorer cognition 6 months after a course of ECT [7]. A recent retrospective study of 10 years of cognitive data collected on patients who underwent ECT found advancing age to be associated with poor cognitive outcomes, though the effect size of this association was attenuated when those aged above 65 were removed to control for those with dementia or early cerebrovascular disease [41]. That study also excluded patients with schizophrenia. Only one previous study has been done on cognition in patients with schizophrenia undergoing ECT, which found no association between age and cognitive change [36]. However, that study had a relatively small sample size of 81, and only compared those with improved MoCA scores to those without. In our study of 125 patients, we looked at predictors of MoCA deterioration as well as improvement, which may explain why an association between advanced age and deteriorating cognition was found. However, this finding warrants further study given the naturalistic design of our study.

A previous study on a population of patients with depression found an association between female sex and cognitive deficits after ECT [7]. This was attributed to the relatively lower seizure threshold found in women compared to men; in this study, the electrical dosage was not moderated based on individual patients’ seizure threshold, and the authors postulated that female patients may have been more likely to receive suprathreshold dosing compared to the males, explaining the greater cognitive deficits. Brus et al in a study published in 2018 also found similar results where females had more cognitive deficits after ECT using formula-based stimulus dosing [42]. In contrast, our study found an association between female sex and improvements in MoCA. This association may be due to our use of dose titration according to seizure threshold rather than formula-based dosing avoiding suprathreshold dosing of female patients.

Our study also found that involuntary admission was independently associated with less deterioration in MoCA. We hypothesize that those who are involuntarily admitted are more unwell and symptomatic, and as such, have a lower baseline cognition which improves after ECT treatment.

Evidence supporting the efficacy of ECT in schizophrenia has increased in recent years, but concerns over its detrimental effects on cognition limit the adoption of ECT in clinical practice. Our study suggests that in patients with schizophrenia or schizoaffective disorder with positive psychotic symptoms, undergoing ECT, poorer baseline cognition predicts cognitive improvement. A possible reason why this finding has not been noted other studies is that patients with low MoCA scores are usually not included in randomized-controlled trials, given their inability to give informed consent [43]. Our previous study provides evidence that the lack of capacity to consent for treatment is associated with poor baseline cognition, and also predicts improvement in cognition with ECT [44]. This current study reinforces that patients with poor baseline cognition show improved cognitive outcomes with ECT, and this may be associated with improvements in negative symptoms. The association between cognition and negative symptom changes will be the subject of a future study. We propose that patients with schizophrenia with impaired cognition at baseline should not be excluded from ECT treatment on the basis of concerns over cognitive impairment. Elderly patients are more likely to experience cognitive impairment with ECT and treatment should utilise memory-sparing ECT techniques (such as seizure-titration) to preserve cognition in this group.

## 5. Limitations

The major limitation of our study is the cognitive assessment with the MoCA scale, which is a brief cognitive screening tool. Some patients may have been too ill to be able to give an appropriate MoCA measurement. We performed a sensitivity analysis in our study, which found that nearly half (48.3%) of patients who were unable to complete the MoCA prior to ECT and were excluded from the primary analysis were subsequently able to complete it post-ECT, suggesting an increased ability to perform standardized cognitive assessments after ECT. It is also important to note that our study was carried out in a naturalistic fashion, including a significant proportion of patients (84%) who were very ill and lacked capacity to consent for treatment [43]. Using more detailed, gold-standard cognitive tests in such a population would be unrealistic. An additional limitation is that our study did not measure for retrograde amnesia, which is the main cognitive side effect associated with ECT [45]. Our study also did not account for other confounding factors such as the premorbid intellectual functioning, presence of neurocognitive disorders, educational level, dosage of medicine and other complimentary psychotherapies, which may also be associated with the cognitive change. Therefore, it is difficult to attribute changes in cognition in our patient groups purely to ECT. However, we noted that patients with clinically reliable cognitive improvements saw a mean 9–10-point improvement in MoCA scores over the course of 10 ECT treatments (3–4 weeks’ duration). In comparison, other interventions aimed specifically at improving cognition in patients with schizophrenia, such as Computer-Assisted Cognitive Remediation therapy, show modest effect sizes over a longer duration of treatment [46, 47]. Medications and other neurostimulation techniques such as Transcranial Magnetic Stimulation have not been associated with any reliable improvements in cognition [48, 49]. It is therefore reasonable to conclude that there are independent effects of ECT on cognitive improvement in these patients. This, however, needs to be confirmed with more detailed neurocognitive testing.

Another limitation is that we do not have data on the usage or discontinuation of medications for patients during the course of ECT. The addition or discontinuation of medications pre, during and post-ECT could impact the cognitive function of the patients, and hence the MoCA scores.

Given that the presence of Schizophrenia is an exclusion criteria for the diagnosis of Mild Cognitive Impairment or Major Neurocognitive Disorder (Criterion D as per DSM V) [5], it is unlikely that there is a significant burden of either of these conditions in our patient population It is more likely that any cognitive impairment seen in our patients at baseline is due to their psychosis. However, it is possible that the older patients in our sample may have had some age related cognitive impairment at baseline, which may explain the association seen between advanced age and worsening cognition with ECT seen in our study. Finally, our database did not record the age of onset of schizophrenia for our patients, which has been shown to be an important predictor of cognitive outcomes [50].

## 6. Conclusion

Our findings suggest that age predicted MoCA deterioration. Lower pre-ECT MoCA and female sex predicted improvement. The use of dose titration instead of a fixed dose could explain MoCA improvement in females seen in our study. Our findings also suggest that low pre-treatment cognition should not be a contraindication to prescribing ECT in schizophrenia.

## Supporting information

S1 Table Predictors of MOCA deterioration in ECT (outliers removed). Abbreviations: ECT–Electroconvulsive Therapy, GAF–Generalized Assessment of Function, BPRS–Brief Psychiatric Rating Scale, MoCA–Montreal Cognitive Assessment *P<0.05.
(DOCX)

S2 Table Predictors of MoCA improvement in ECT (outliers removed). Abbreviations: ECT–Electroconvulsive Therapy, GAF–Generalized Assessment of Function, BPRS–Brief Psychiatric Rating Scale, MoCA–Montreal Cognitive Assessment *P<0.05. ** P<0.001.
(DOCX)
