# Supplementary material for: Predictors of cognitive changes in patients with schizophrenia undergoing electroconvulsive therapy
Source: PLoS One. 2023 May 9;18(5):e0284579. doi: 10.1371/journal.pone.0284579 (PMC10168561; doi:10.1371/journal.pone.0284579)
Supplement: S1 Table — Abbreviations: ECT–Electroconvulsive Therapy, GAF–Generalized Assessment of Function, BPRS–Brief Psychiatric Rating Scale, MoCA–Montreal Cognitive Assessment *P<0.05. (DOCX) [file pone.0284579.s001.docx]

**Supplementary Table 1 - Predictors of MOCA deterioration in ECT (outliers removed)**

| **Outcome** | **Risk predictor** | | **Crude** | | | | | **Adjusted** | | | | |
| --- | --- | --- | --- | --- | --- | --- | --- | --- | --- | --- | --- | --- |
|  |  |  | **B** | **OR** | **95% Cl for OR** | | **P value** | **B** | **OR** | **95% CI for OR** | | **P value** |
|  |  |  |  |  | **Lower Bound** | **Upper Bound** |  |  |  | **Lower Bound** | **Upper Bound** |  |
| MOCA deterioration vs MOCA no change | Age | >55 years | 1.723 | 5.600 | 1.118 | 28.047 | **0.036*** | 2.44 | 11.43 | 1.11 | 117.90 | **0.041*** |
|  |  | ≤55 years | Ref. | | | | | | | | | |
|  | No. ECT | | -0.11 | 0.9 | 0.76 | 1.07 | 0.225 | -0.26 | 0.77 | 0.55 | 1.08 | 0.129 |
|  | MoCA pre-ECT | | -0.1 | 0.9 | 0.81 | 1 | 0.052 | -0.05 | 0.95 | 0.82 | 1.11 | 0.506 |
|  | GAF pre-ECT | | -0.06 | 0.94 | 0.86 | 1.02 | 0.146 | -0.03 | 0.97 | 0.84 | 1.12 | 0.652 |
|  | BPRS pre-ECT | | 0.02 | 1.02 | 0.97 | 1.07 | 0.408 | -0.01 | 0.99 | 0.92 | 1.07 | 0.843 |
|  | Gender | Female | 1.1 | 3 | 1.09 | 8.29 | **0.034*** | 1.27 | 3.58 | 0.84 | 15.29 | 0.085 |
|  |  | Male | Ref. | | | | | | | | | |
|  | Admission status | Involuntary | -0.84 | 0.43 | 0.15 | 1.23 | 0.116 | -1.77 | 0.17 | 0.03 | 0.97 | **0.046*** |
|  |  | Voluntary | Ref. | | | | | | | | | |
|  | Consent | By others | 0.9 | 2.47 | 0.64 | 9.46 | 0.188 | 1.20 | 3.31 | 0.32 | 34.57 | 0.317 |
|  |  | By self | Ref. | | | | | | | | | |
|  | Antidepressants | YES | -0.38 | 0.69 | 0.25 | 1.91 | 0.472 | -0.45 | 0.64 | 0.09 | 4.52 | 0.654 |
|  |  | NO | Ref. | | | | | | | | | |
|  | Lithium | YES | -1.33 | 0.27 | 0.03 | 2.69 | 0.261 | -3.06 | 0.05 | 0.00 | 1.24 | 0.067 |
|  |  | NO | Ref. | | | | | | | | | |
|  | Benzodiazepines | YES | 0.17 | 1.19 | 0.45 | 3.15 | 0.73 | 1.40 | 4.04 | 0.88 | 18.51 | 0.073 |
|  |  | NO | Ref. | | | | | | | | | |
|  | Anticonvulsants | YES | -1.2 | 0.3 | 0.08 | 1.11 | 0.071 | -0.10 | 0.91 | 0.10 | 7.95 | 0.931 |
|  |  | NO | Ref. | | | | | | | | | |
|  | Clozapine | YES - with no/minimal response | 0.29 | 1.34 | 0.37 | 4.86 | 0.657 | 1.19 | 3.27 | 0.40 | 26.86 | 0.269 |
|  |  | YES - with partial response | 0.24 | 1.28 | 0.26 | 6.36 | 0.767 | -0.62 | 0.54 | 0.05 | 5.71 | 0.605 |
|  |  | NO | Ref. | | | | | | | | | |

*Abbreviations: ECT – Electroconvulsive Therapy, GAF – Generalized Assessment of Function, BPRS – Brief Psychiatric Rating Scale, MoCA – Montreal Cognitive Assessment*

**P<0.05*
