# Supplementary material for: Predictors of cognitive changes in patients with schizophrenia undergoing electroconvulsive therapy
Source: PLoS One. 2023 May 9;18(5):e0284579. doi: 10.1371/journal.pone.0284579 (PMC10168561; doi:10.1371/journal.pone.0284579)
Supplement: S2 Table — Abbreviations: ECT–Electroconvulsive Therapy, GAF–Generalized Assessment of Function, BPRS–Brief Psychiatric Rating Scale, MoCA–Montreal Cognitive Assessment *P<0.05. ** P<0.001. (DOCX) [file pone.0284579.s002.docx]

**Supplementary Table 2 - Predictors of MoCA improvement in ECT (outliers removed)**

| **Outcome** | **Risk predictor** | | **Crude** | | | | | **Adjusted** | | | | |
| --- | --- | --- | --- | --- | --- | --- | --- | --- | --- | --- | --- | --- |
|  |  |  | **B** | **OR** | **95% Cl for OR** | | P value | **B** | **OR** | **95% CI for OR** | | **P value** |
|  |  |  |  |  | **Lower Bound** | **Upper Bound** |  |  |  | **Lower Bound** | **Upper Bound** |  |
| MOCA improvement vs MOCA no change | Age | >55 years | 1.09 | 2.98 | 0.61 | 14.59 | 0.178 | 0.96 | 2.61 | 0.21 | 32.78 | 0.457 |
|  |  | ≤55 years | Ref. | | | | | | | | |  |
|  | No. ECT | | -0.03 | 0.97 | 0.83 | 1.13 | 0.681 | -0.23 | 0.79 | 0.57 | 1.11 | 0.176 |
|  | MoCA pre-ECT | | -0.25 | 0.78 | 0.70 | 0.87 | **<0.001**** | -0.27 | 0.76 | 0.65 | 0.89 | **0.001*** |
|  | GAF pre-ECT | | -0.06 | 0.94 | 0.87 | 1.02 | 0.119 | -0.09 | 0.92 | 0.79 | 1.05 | 0.221 |
|  | BPRS pre-ECT | | 0.03 | 1.03 | 0.99 | 1.08 | 0.153 | 0.03 | 1.03 | 0.95 | 1.13 | 0.450 |
|  | Gender | Female | 1.47 | 4.33 | 1.69 | 11.12 | **0.002*** | 2.58 | 13.24 | 1.84 | 95.43 | **0.010*** |
|  |  | Male | Ref. | | | | | | | | | |
|  | Admission status | Involuntary | 0.31 | 1.36 | 0.49 | 3.82 | 0.555 | -0.28 | 0.75 | 0.10 | 5.45 | 0.779 |
|  |  | Voluntary | Ref. | | | | | | | | | |
|  | Consent | By others | 0.96 | 2.60 | 0.78 | 8.64 | 0.119 | -1.20 | 0.30 | 0.02 | 4.30 | 0.376 |
|  |  | By self | Ref. | | | | | | | | | |
|  | Antidepressants | YES | -0.92 | 0.40 | 0.15 | 1.05 | 0.064 | -0.60 | 0.55 | 0.07 | 4.02 | 0.554 |
|  |  | NO | Ref. | | | | | | | | | |
|  | Lithium | YES | 0.06 | 1.06 | 0.25 | 4.57 | 0.939 | -0.07 | 0.93 | 0.06 | 14.68 | 0.959 |
|  |  | NO | Ref. | | | | | | | | | |
|  | Benzodiazepines | YES | 0.32 | 1.38 | 0.57 | 3.34 | 0.482 | 0.12 | 1.13 | 0.20 | 6.37 | 0.891 |
|  |  | NO | Ref. | | | | | | | | | |
|  | Anticonvulsants | YES | -0.47 | 0.62 | 0.23 | 1.70 | 0.356 | 0.89 | 2.43 | 0.30 | 19.66 | 0.406 |
|  |  | NO | Ref. | | | | | | | | | |
|  | Clozapine | YES - with no/minimal response | 0.24 | 1.27 | 0.39 | 4.15 | 0.688 | 1.32 | 3.76 | 0.36 | 38.82 | 0.266 |
|  |  | YES - with partial response | 0.43 | 1.54 | 0.37 | 6.43 | 0.551 | -0.30 | 0.74 | 0.08 | 7.24 | 0.796 |
|  |  | NO | Ref. | | | | | | | | | |

*Abbreviations: ECT – Electroconvulsive Therapy, GAF – Generalized Assessment of Function, BPRS – Brief Psychiatric Rating Scale, MoCA – Montreal Cognitive Assessment*

**P<0.05*

*** P<0.001*
